# Mechanisms and Therapeutic Targets of Cardiac Regeneration: Closing the Age Gap

**DOI:** 10.3389/fcvm.2018.00007

**Published:** 2018-02-05

**Authors:** Raphael F. P. Castellan, Marco Meloni

**Affiliations:** ^1^British Heart Foundation and University of Edinburgh Centre for Cardiovascular Science, Queen’s Medical Research Institute, Edinburgh, United Kingdom

**Keywords:** cardiac regeneration, mechanisms, therapeutic, targets, aging

## Abstract

While a regenerative response is limited in the mammalian adult heart, it has been recently shown that the neonatal mammalian heart possesses a marked but transient capacity for regeneration after cardiac injury, including myocardial infarction. These findings evidence that the mammalian heart still retains a regenerative capacity and highlights the concept that the expression of distinct molecular switches (that activate or inhibit cellular mechanisms regulating tissue development and regeneration) vary during different stages of life, indicating that cardiac regeneration is an age-dependent process. Thus, understanding the mechanisms underpinning regeneration in the neonatal-infarcted heart is crucial to develop new treatments aimed at improving cardiovascular regeneration in the adult. The present review summarizes the current knowledge on the pathways and factors that are known to determine cardiac regeneration in the neonatal-infarcted heart. In particular, we will focus on the effects of microRNA manipulation in regulating cardiomyocyte proliferation and regeneration, as well as on the role of the Hippo signaling pathway and Meis1 in the regenerative response of the neonatal-infarcted heart. We will also briefly comment on the role of macrophages in scar formation of the adult-infarcted heart or their contribution for scar-free regeneration of the neonatal mouse heart after myocardial infarction. Although additional research is needed in order to identify other factors that regulate cardiovascular regeneration, these pathways represent potential therapeutic targets for rejuvenation of aging hearts and for improving regeneration of the adult-infarcted heart.

Cardiac remodeling and subsequent heart failure (HF) remain critical issues after myocardial infarction (MI). Following MI, the adult mammalian myocardium undergoes a complex remodeling process initiated by the death of billions of cells within the ischemic region (1). Those are mostly cardiomyocytes, endothelial cells, and fibroblasts. The adult mammalian-infarcted heart fails to regenerate the lost tissue. Instead, damaged myocardium is replaced by fibrotic tissue, leading to altered contractile function and eventually HF (2–4). The World Health Organization and the European Cardiovascular Disease Statistics indicate that cardiovascular disease causes over 4 million deaths in Europe each year, and more than 40% of these deaths are attributable to MI. Due to the considerable improvement in reperfusion techniques the survival rate of patients following MI has been dramatically improved (5, 6). However, HF is a frequent complication of MI and remains a major cause of morbidity and mortality worldwide (7). Indeed, within 5 years of first MI, 16% of men and 22% of women over 45 years of age will develop HF, and the incidence and prevalence of HF significantly increases in the elderly population (8, 9). With increasing survival post-MI and an aging population, the prevalence of HF is expected to have risen 48% between 2012 and 2030 (8). Despite improvements in conventional treatments aimed at improving or re-establishing the revascularization of the infarcted myocardium (such as coronary artery bypass surgery or percutaneous coronary intervention), current therapies are limited (10, 11) and do not lead to regeneration of the lost tissue. There is therefore an urgent need for novel therapies for the treatment of MI.

In this context, recent research has shown that whereas a regenerative response is limited in the adult mammalian heart, the adult zebrafish is able to regenerate lost or damaged cardiac tissue (12–15). Most importantly, additional studies have recently demonstrated that the neonatal mouse heart can fully regenerate after resection of the left ventricular (LV) apex, and after induction of MI at early stages (postnatal day 1, P1) (16–19). However, this regenerative capacity is only transient and is rapidly lost by 7 days of age (18), indicating the existence of a “regenerative window” within the first week after birth in mice. Remarkably, newborn humans may also possess the intrinsic capacity to repair their heart after MI (19). Haubner et al. have recently observed full cardiac functional recovery within weeks after the occurrence of MI in a newborn child (19). While suggesting that the mammalian heart is able of regeneration, those findings also highlight the age-dependency of this process. This highlights the concept that the expression of distinct molecular switches activating or inhibiting cellular mechanisms regulating tissue regeneration vary during different stages of life. Thus, a better understanding of the mechanisms that regulate complete cardiac repair and regeneration in the neonatal-infarcted heart could provide the basis for novel therapeutic approaches for cardiovascular regeneration following MI in the adult. This review illustrates the current understanding of the most important mechanisms that regulate cardiac regeneration in the neonatal mammalian heart and discusses the potential therapeutic targets that can be used in order to rejuvenate the adult-infarcted heart and improve its cardiac regeneration following MI.

## Cardiac Regeneration: Evolution and Development

### Cardiac Regeneration in Lower Vertebrates

Organ regeneration has been the focus of scientific research for over a century. For example, the ability of salamanders to regrow an entire lost limb after amputation has been studied for decades and their capacity to regenerate also other damaged parts of their bodies is still of interest in the field of regenerative medicine. Cardiac regeneration was however only recently investigated formally in lower vertebrate models. Zebrafish imposed itself as a standard model of cardiac regeneration as a result of its straightforward genetic manipulation and short generation time (20, 21).

Zebrafish (*Danio rerio*), a member of the teleost infraclass of bony fish, was first reported to harbor potent cardiac regenerative capacities by Poss et al. (12). After surgical removal of about 20% of the ventricular myocardium of adult fish, the authors initially observed a rapid formation of a blood clot. This was replaced by a fibrin cap by 9 days post-resection. Over the next 60 days, the fibrin cap was shown to regress as cardiomyocytes replaced the resected area, resulting in the complete regeneration of the myocardium. Using this resection model, the authors were also able to show evidence of cardiomyocyte proliferation and suggested that cell proliferation was essential to scar-free regeneration of the adult zebrafish heart. Subsequently, other studies using lineage tracing and electron microscopy techniques demonstrated that cardiomyocyte replenishment of the resected myocardium in the adult zebrafish occurred through existing cardiomyocyte de-differentiation and subsequent proliferation (15).

### Cardiac Regeneration in Mammals

Mammalian models of cardiac regeneration were non-existent until the description by Porrello et al. that P1-old neonatal mice could fully regenerate their myocardium by 21 days following apical resection (18). Subsequently, Porrello et al. (17) and Haubner et al. (16) developed a model of MI in P1-old mice by ligation of the left coronary artery. Similarly to what observed with the resection model, neonatal mice could regenerate the injured myocardium by 21 days following MI at P1. This was also accompanied by full recovery of cardiac function. Importantly, this regenerative capacity is only transient and is strictly limited to the first week after birth. In fact, neonatal mice undergoing MI at P7 were unable to regenerate the lost myocardium and instead formed a scar, similar to the adult heart (17).

Myocardial infarction in P1 mice was associated with increased markers of cell death (such as cleaved caspase 3), functional decrease in the days following injury, and decreased proportion of viable myocardium (16, 17). At day 3 post-MI, observed extensive cardiomyocyte loss and immune cell infiltration were observed (17). By day 7, a scar had formed within the injured myocardium. This scar was then gradually removed as the myocardium regenerated leading to minimal interstitial fibrosis by day 21 post-MI. Similar to the zebrafish, neonatal mice were shown to achieve cardiac regeneration through de-differentiation and proliferation of cardiomyocytes, rather than through a stem-cell-based mechanism (16–18).

It is commonly believed that following injury, developmental pathways such as Wnt, Notch, and Hedgehog are re-activated in an effort to restore tissue integrity (22, 23). It is however very clear that successful regeneration is both age-dependent and organ-specific (12, 17, 24). In this context, the existence of a “regenerative window” within the first week after birth (at least in mice) support the idea that the post-natal heart still harbors regenerative capacities that could be promoted after injury to improve the regenerative potential of the adult-infarcted heart and reduce the burden of MI.

### The (Limited) Regenerative Capacity of the Human-Infarcted Heart

Even though the adult mammalian heart clearly lacks potent regenerative capacities, a study by Bergmann et al. showed that the adult human heart was able of slow cardiomyocyte renewal throughout life (25). This suggests that the adult mammalian heart retains some regenerative capacities that may be stimulated to improve the outcome following MI.

In contrast to the adult, newborn humans and infants seem to still harbor potent cardiac regenerative capacities. Indeed, a study by Fratz et al. reported very little myocardial scarring and normal function in adults having had cardiac corrective surgery for anomalous origin of the left coronary artery from the pulmonary artery (ALPACA) as children (26). Moreover, in a recent case report Haubner et al. have recently observed full cardiac recovery within weeks after the occurrence of MI in a newborn child (19).

## Mechanisms of Cardiac Regeneration and Potential Therapeutic Targets

Achieving successful cardiac regeneration necessitate three key phenomena: cardiomyocyte replenishment, removal of interstitial fibrosis, and revascularization of the regenerated myocardium. Since the discovery of the neonatal mouse regenerative capacities, much work has focused on understanding the mechanisms behind it in the aim to identify targets to promote regeneration over scar formation in the adult myocardium following MI. Cardiomyocyte replenishment following neonatal MI is considered as the step limiting factor in achieving cardiac regeneration. To date, most of the research has therefore focused on the regulation of neonatal cardiomyocyte proliferation, and has identified several pathways and factors that are involved in the regulation of cardiomyocyte proliferation and that can be considered as potential therapeutic targets for improving cardiac regeneration in the adult-infarcted heart.

### MicroRNAs (miRNAs)

Recent findings demonstrate that the regulation of cardiovascular development and regeneration is mediated by non-coding RNAs known as miRNAs (27–31). miRNAs are inhibitory regulators of gene expression, which act by binding to complementary mRNA transcripts for promoting either their degradation or translational repression (32). The expression of many miRNAs vary during different stages of life, reflecting their role as molecular switches that activate or inhibit cellular mechanisms that regulate cell and tissue development (33), including heart development (34).

In the neonatal mouse heart miRNAs have been linked to cardiomyocyte binucleation and cell cycle exit (35). miRNA expression profile performed in mouse cardiac ventricles isolated from P1 (within the regenerative window) and P10 (beyond the regenerative window) mice showed that 71 miRNAs are either up or downregulated during these developmental stages (35).

Notably, several large miRNA families (such as the miRNA-15, miRNA-30, and let-7 families) were upregulated in P10 cardiac ventricles, and miRNA-195 (a member of the miRNA-15 family) was shown to be the most highly upregulated miRNA. Cardiomyocyte-targeted overexpression of miRNA-195 was associated with cardiac developmental defects and reduced cardiomyocyte proliferation in P1 mice (35). In addition, cardiomyocyte-directed overexpression of miRNA-195 prevented cardiac regeneration of the neonatal mouse heart after induction of MI at P1. This was associated with decreased cardiomyocyte proliferation at day 7 post-MI, and impaired functional recovery and cardiomyocyte hypertrophy at day 21 post-MI (17).

Other miRNAs have been shown to regulate cardiomyocyte proliferation and regeneration. Eulalio et al. (36) performed a high-throughput functional screening to assess the miRNAs capable of promoting cardiomyocytes proliferation. They found that at least 40 miRNAs increased both DNA synthesis and cytokinesis in neonatal mouse and rat cardiomyocytes. miRNA-590 and miRNA-199 were further tested and showed to promote cell cycle re-entry of adult cardiomyocytes *ex vivo*. In addition, overexpression of either miRNA-199 or miRNA-590 in the heart of adult mice undergoing MI showed that both miRNAs induced cardiac regeneration and improved cardiac function (36).

Another study showed the involvement of the miRNA-17-92 cluster in cardiomyocytes proliferation of postnatal and adult hearts (37). Nkx.2.5-mediated cardiac specific deletion of the miRNA-17-92 cluster resulted in partial embryonic lethality, and the miRNA-17-92 KO mice that survived had a smaller heart and a marked reduction of cardiomyocyte proliferation compared with littermate controls. On the other hand, cardiac specific conditional transgenic mice overexpressing the miRNA-17-92 cluster were characterized by a significant increased proliferation of cardiomyocytes during either embryonic or postnatal stages. Moreover, these mice showed a higher heart/body weight ratio and increased thickness of the ventricular wall. Additionally, induction of MI in adult transgenic mice overexpressing the miRNA-17-92 cluster resulted in the reduction of the cardiac scar size, increased cardiomyocyte proliferation, and improvement of cardiac function (37).

The miRNA-34a has also been demonstrated to regulate cell-cycle activity and death in cardiomyocytes (38). miRNA-34a expression in the heart is low in the early-postnatal period and it increases after P8 (beyond the regenerative window), and whereas overexpression of the miRNA-34a prevented cardiac regeneration in the neonatal mouse-infarcted heart, its inhibition improved cardiac function and repair in the adult-infarcted heart through the modulation of cell cycle and survival genes such as Bcl2, Cyclin D1, and Sirt1 (38).

### The Hippo Signaling Pathway

The Hippo pathway has been demonstrated to be critically involved in cardiac development and regeneration and may represent a promising therapeutic target in the setting of MI. The pathway consists of a cascade of kinases controlling cell proliferation and organ size. Activation of the Hippo pathway leads to the phosphorylation of the transcriptional co-activators yes associated protein (YAP) and WW domain-containing transcription regulator protein 1 (TAZ). This prevents their nuclear localization and, in the fetal mouse heart, restrains cardiomyocyte proliferation (39, 40). Inactivation of the pathway indeed leads to largely overgrown hearts with increased cardiomyocyte proliferation (40). Conversely, cardiac specific deletion of YAP is associated with decreased cardiomyocyte proliferation at P1 and the development of dilated cardiomyopathy in adults.

The Hippo/YAP axis also plays crucial roles following cardiac injury in both neonatal and adult mice. Cardiac specific deletion of YAP abrogates the regenerative response of neonatal mice leading to the formation a fibrotic scar and functional impairment by day 27 post-MI. On the other hand, stabilization of YAP promotes proliferation of cardiomyocytes following injury in mice at P28, thus outside of the regenerative window, suggesting potential for the pathway to be manipulated to unlock cardiac regenerative capacities in the adult mammal. The Hippo pathway therefore represents a key modulator of cardiac regeneration and more specifically cardiomyocyte replenishment.

Due to the emerging role of the pathway in controlling organ growth and size as well as its tumor suppressor capacities, pharmacological manipulation of the pathway has been the focus of a number of studies (41). Of interest, the small molecule inhibitor 9E1 has shown promising *in vitro* results in targeting mammalian STE20-like protein kinases (MSTs); kinases involved in the phosphorylation and thereby repression of YAP/TAZ. The use of such inhibitor *in vivo* is yet to be tested but may represent an avenue for the manipulation of the Hippo pathway with the aim to unlock cardiac regenerative capacities in the adult mammalian heart. Recently, deletion of the Hippo pathway component Salvador has been shown to increase vascularity and reduce fibrosis in the mouse-infarcted heart, leading to improved cardiac function (42). Moreover, gene therapy-mediated knockdown of Salvador in cardiomyocytes induced their proliferation improving cardiac function after MI, thus confirming the Hippo pathway as potential therapeutic target after MI.

### Meis1

Meis1 belongs to the TALE family of homeodomain transcription factor and is well known for its role in hematopoiesis (43). Meis1 has been also shown to be essential for cardiac development (44, 45) and has recently been identified as being critical for postnatal cardiomyocyte proliferation (46). Cardiomyocyte-specific deletion of *Meis1* resulted in increased cardiomyocyte proliferation and increased percentage of mononucleated cardiomyocytes postnatally (P14, beyond the regenerative window). On the other hand, induction of MI in P1-old cardiac-specific *Meis1* overexpressing mice resulted in premature cell-cycle arrest and inhibition of cardiac regeneration, thus suggesting a role for *Meis1* in cell-cycle activity and cardiomyocytes nucleation. Interestingly, miRNA-548c-3p, miRNA-509-3p, and miRNA23b-3p were shown to induce significant proliferation of adult rat cardiomyocytes through translational inhibition of Meis1 (47). Moreover, it was also recently shown that Tbx20 (a member of the Tbx1 subfamily of T-box genes which is required for cardiomyocyte proliferation during heart development) (48) can act as transcriptional repressor of Mes1 leading to increased proliferation of cardiomyocytes and preservation of cardiac function following MI in adult mice (49), thus confirming the key role of Meis1 in regulating cardiomyocyte proliferation and its promising potential as therapeutic target for cardiac regeneration post-MI.

### Immune Response Signals

The immune system is actively involved in tissue growth during development and participates to tissue repair and regeneration after injury (including MI) (50–53). Following MI, necrotic cell death triggers an immune response characterized by the activation of inflammatory cells (mainly monocytes/macrophages) responsible for removing dead cells and debris, and for secreting cytokines and factors in order to attempt to restore the integrity of the injured heart (50–54). In the adult-infarcted heart this immune response participates in the activation of fibroblasts and proliferation of endothelial cells (55, 56). This leads to the formation of a fibrotic scar, thus preventing the rupture of the ventricular wall but contributing to a progressive impairment of cardiac function which eventually leads to HF (57).

On the other hand, macrophages are required for scar-free regeneration of the neonatal mouse heart following MI (53, 58). Interestingly, neonatal mice mobilize a subset of tissue-resident macrophages of yolk-sac origin immediately following genetic ablation of cardiomyocytes, whereas adult mice preferentially expand a population of bone-marrow-derived macrophages (59). Cardiac yolk-sac-derived macrophages have increased angiogenic capacities *in vitro* as compared with adult bone-marrow-derived macrophages (59). Such differences in the immune response and the intrinsic capacities of the cells involved may therefore participate in the regenerative capacities of the neonatal mouse (60). Recently, Quaife-Ryan et al. (61) published an extensive report on the transcriptional differences between neonatal and adult leukocytes following MI which may contribute to a regenerative or fibrotic phenotype. This study sets a stepping stone to in-depth characterization of the pathways involved during regeneration vs. fibrotic repair. Further studies should investigate the roles and interplay of the different leukocyte population during regeneration and fibrotic repair in order to identify targets for the promotion of regeneration. More information about the importance of immune modulation if cardiac repair and regeneration can be found in Zlatanova et al. (58).

### Cardiac-Resident Stem/Progenitor Cells

The adult mouse heart possesses a resident population of c-kit^+^/Lin^−^ cells expressing markers of cardiomyocyte progenitors with clonogenic, self-renewing capacities and able to differentiate into several cell types including cardiomyocytes (62), and the c-kit^+^ cell population has been shown to act as cardiomyocyte progenitors in the adult mouse following injury (63). Interestingly, also the human heart contains a population of c-kit^+^ cardiac-resident stem cells that can divide and differentiate into myocytes (64), but their regenerative potential decreases in the setting of chronic HF and is associated with decreased telomerase activity and cellular senescence (64). However, c-kit is expressed by a heterogeneous population (65, 66) and only a very small fraction of c-kit^+^ cells has the ability to differentiate into cardiomyocytes (66). In this context, however, although regeneration of the neonatal heart has been proposed to occur through de-differentiation of cardiomyocytes, the role (if any) of resident cardiac stem/progenitor cells in the neonatal heart regeneration has not been investigated.

## Conclusion

The regeneration potential of essentially all tissues and organs, including the heart, is known to decrease during the aging process. The observation that the neonatal mouse heart possesses potent regenerative capacities has provided the field of cardiovascular regeneration with a strong model for the identification of pathways and therapeutic targets for the promotion of regeneration over fibrotic repair following MI. As outlined in this review, a number of pathways and factors that regulate cardiovascular regeneration of the neonatal heart have been identified. These pathways represent potential therapeutic targets for rejuvenation of aging hearts and for improving regeneration of the adult-infarcted heart.

## Author Contributions

RC and MM performed literature search and wrote the manuscript.

## Conflict of Interest Statement

The authors declare that the research was conducted in the absence of any commercial or financial relationships that could be construed as a potential conflict of interest.
